# Participation of ethnic minorities in Parkinson’s research: challenges and needs. A qualitative study

**DOI:** 10.1093/ageing/afaf296

**Published:** 2025-10-24

**Authors:** Mouhammed Ramadhan, Mahil Tufail, Joshua Stott, Anette Schrag

**Affiliations:** Department of Clinical and Movement Neurosciences, University College London, London, WC1E 6BT, UK; Department of Clinical and Movement Neurosciences, University College London, London, WC1E 6BT, UK; Department of Clinical, Educational and Health Psychology, University College London, London, UK; Department of Clinical and Movement Neurosciences, University College London, London, WC1E 6BT, UK

**Keywords:** barriers, participation, Parkinson’s disease, improve, ethnic minority, qualitative research, older people

## Abstract

**Objective:**

To explore barriers to participation in Parkinson’s disease (PD) research trials amongst ethnic minority (EM) individuals in the UK and to identify potential strategies to improve inclusivity.

**Design:**

A qualitative study using semi-structured interviews and thematic analysis.

**Setting:**

Participants were recruited primarily through community outreach in the UK.

**Participants:**

Twenty-one individuals diagnosed with PD, self-identifying as belonging to EM groups, participated in the study. The sample included individuals from South Asian, Black African and Middle Eastern backgrounds.

**Results:**

Five themes were identified: (i) Lack of Awareness of Research Opportunities; (ii) Mistrust and Misconceptions about Research, where fears and misunderstandings about research processes contributed to hesitancy; (iii) Understanding the importance and scope of research, some participants viewed research only as a means to find a cure, while others emphasised the need for studies on non-motor symptoms; (iv) Practical and Parkinson’s-related barriers, including fatigue, travel difficulties, financial constraints and language barriers; and (v) Facilitators to Participation many preferring flexible and remote participation options.

**Conclusions:**

This study found addressing barriers to participation requires tailored engagement strategies, transparent communication, diverse representation in research teams and practical support measures. Emphasising the importance of research and its potential to improve treatments and outcomes is essential to improving inclusivity and accessibility in PD research.

## Key Points

- Practical and Parkinson’s-related barriers, such as fatigue, mobility issues and time constraints limit participation.
- Ethnic minority individuals with Parkinson’s face significant barriers to research participation.
- Mistrust, stigma and low awareness reduce engagement.

## Introduction

Parkinson’s disease (PD) affects individuals worldwide, with an estimated 6.1 million individuals in 2016 worldwide having PD and a projected prevalence of 14.2 million by 2040 [1]. Despite this, most research focuses on predominantly White populations, limiting generalizability [2]. A 2025 literature review by Siddiqi *et al.* found that only 4.8% of 1142 PD studies published between 2000 and 2024 included race or ethnicity as part of their analysis, demonstrating a significant underrepresentation of diverse populations in PD research [2].

The reported prevalence of PD varies by country and ethnic group around the world and, in addition to differences in longevity [3], a variety factors including genetic and environmental factors are likely to be responsible. In the UK, a report estimated that there are around 145 000 people with PD in the UK [4], but noted that there is insufficient information on prevalence by ethnicity due to inconsistent recording in primary care data. A large UK cohort study [5], also found that ethnicity could not be analysed due to substantial missing data in primary care records, limiting epidemiological insight into the ethnic composition of people with Parkinson’s (PwP) in the UK. In a US study [6] reported notable ethnic differences in PD prevalence between 2168 per 100 000 in White individuals, 1036 in Black individuals, and 1138 in Asian individuals. Other US studies [7], however, have not found such disparities in PD prevalence in the USA, and suggested that these differences in prevalence may reflect healthcare access disparities rather than true biological differences, as Black individuals were twice as likely to be previously undiagnosed with parkinsonism in a population-based US study [8]. These conflicting findings reflect the paucity of research in ethnic minorities with ongoing underrepresentation of ethnic minority (EM) groups, limiting understanding of PD in people with different ethnicities.

A systematic review of PD clinical trials conducted over a 20-year period in the USA revealed that only 17% of the 239 studies reported racial and ethnic participation [9]. The review included a diverse range of studies, such as the NIH Exploratory Trials in PD, therapeutic drug trials and quality of life assessments. Only nine studies reported detailed breakdowns of ethnicity enrolment numbers for African American, Hispanic, Asian and other minority groups. African American and Hispanic participants each constituted <2% of total enrolment across these studies. Most barriers seemed to occur before screening, likely due to limited clinic access, fewer specialist referrals and low awareness of research opportunities.

In those trials, non-White participants represented ~8% of participants, whereas non-White individuals made up ~20% of the US population aged 60 and over at that time [9], which limits the applicability of research to non-White populations. This research under-representation mirrors the clinical picture in PD, where ethnic minorities are less likely to seek clinical care and experience delayed diagnoses, and have lower treatment rates [10, 11]. Additionally, ethnic differences in disease progression and treatment response have been reported [12].

While the underrepresentation of ethnic minorities in PD research is well-documented [9], the specific barriers influencing their participation require further research. PD-specific research like the FIRE-UP PD study has identified obstacles related to infrastructure, study design, participant characteristics and community factors [13]. However, detailed qualitative insights into lived experiences within diverse UK EM communities remain limited. Existing literature often identifies barriers at a general level [9], but does not include the personal narratives and cultural factors influencing research participation decisions.

The specific barriers and facilitators influencing research participation amongst EM individuals with PD, especially from Asian and Arab populations, in European contexts are still not well understood. To our knowledge the only previous work examining barriers to research was conducted in the USA in African American and Hispanic populations. Therefore, the primary aim of this qualitative study was to identify barriers and potential strategies to increase participation in PD research trials amongst various EM groups in the UK.

## Methods

Ethics approval for this study was granted by the Yorkshire & The Humber–Bradford Leeds Research Ethics Committee. All participants provided written informed consent via REDCap prior to their involvement in the study. The reporting of this qualitative study adheres to the criteria for reporting qualitative research guidelines [14].

### Study design

This was a qualitative study using semi-structured interviews to explore barriers and facilitators to research participation amongst PwP from EM backgrounds in the UK. We used an inductive approach, following Braun and Clarke’s reflexive thematic analysis framework [15], to ensure that themes emerged directly from participants’ lived experiences and perceptions. Interviews were primarily conducted in English, with some responses offered in Urdu to facilitate expression for some participants. Ethnicity was self-reported using the Office for National Statistics ethnic group categories. For full demographic details, see Table 1.

**Table 1:** Participant characteristics.

Age (years), mean ± SD	54 ± 10.67
Sex	
Female, n (%)	8 (38%)
Male, n (%)	13 (62%)
Ethnicity	
Asian/Asian British–Indian	9
Asian/Asian British–Pakistani	6
Other EM groups (e.g. Sri Lankan, Arab, Black African, Mixed)	6
Unemployed, n (%)	1 (5)
Retired, n (%)	7 (35)
Highest qualification	
Secondary education (e.g. GCSE, O-Level, A-Level), n (%)	3 (15)
Higher education (e.g. University degree, vocational course), n (%)	16 (80)
No qualifications, n (%)	1 (5)
Age leaving education (years), mean ± SD	23.7 ± 6.3
Duration of PD (years since diagnosis), mean ± SD	
Dominant hand	
Left, n (%)	3 (15)
Right, n (%)	17 (85)
Marital status	
Married, n (%)	17 (85)
Widowed, n (%)	1 (5)
Separated, n (%)	1 (5)
Single, n (%)	1 (5)
Living situation	
Living with family, n (%)	19 (95)
Living alone, n (%)	1 (5)
Living in	
City, n (%)	13 (65)
Major conurbation, n (%)	3 (15)
Town, n (%)	1 (5)
Number of people took part in previous research studies	2

### Setting

Data collection occurred between December 2022 and January 2024. The interviews were conducted remotely via Zoom and Teams, and the interview questions are provided in Appendix 2 (Supplementary Data). This remote approach was chosen to maximise accessibility for participants across different locations in the UK, accommodating potential mobility challenges associated with PD and in consideration of ongoing public health advisories like COVID-19 during part of the recruitment period.

### Sampling and recruitment

A purposive sampling was used to recruit participants from neurology clinics and community groups. We aimed to capture a diverse range of ethnic backgrounds. The sample size was guided by thematic saturation with recruitment continuing until no new themes emerged and data became repetitive. We initially attempted to recruit through neurology clinics, which yielded limited participation. While we did not specifically explore these reasons but have speculated on this being due to lack of time to explain research in clinic settings. With input from our Patient and Public Involvement and Engagement (PPIE) group, we publicised the study through information at local community centres and support groups for PwP, social media via platforms like (Facebook, Twitter, Instagram), community centres, and snowball sampling (word of mouth, family referrals). Overall, 48 individuals who expressed an initial interest, of whom 21 agreed to participate in the interview study. Further details on recruitment sources and reasons for nonparticipation are provided in Table 4 and Appendix 1 (Supplementary Data). Potential biases arising from our recruitment strategies have been explicitly addressed in the limitations section.

### Analysis

Interviews were recorded and transcribed verbatim using NVivo 14. Data were analysed thematically using Braun and Clarke’s reflexive approach [15], focusing on identifying themes across the dataset. This method involved data familiarisation, coding, theme searching, reviewing and defining themes using an inductive approach. Themes were refined through consultation with co-authors and a self-reflective journal maintained by the first author to ensure rigour and acknowledge biases [16].

### Cultural sensitivity approach

Given the focus on EM participants we used several measures to ensure cultural sensitivity throughout the research process:

During the study design the research team included members from diverse ethnic backgrounds and Interview guides were reviewed for cultural appropriateness. During the data collection phase interview questions were strategically ordered and adapted to avoid culturally sensitive topics initially, building rapport before addressing more personal barriers. Where possible, interviews were conducted in participants preferred language and flexible scheduling accommodated cultural/religious practices. We used open-ended questions that did not make assumptions about participants backgrounds. The interviews started with easier topics and gradually moved to more personal ones, allowing participants to describe their own cultural experiences and beliefs in their own words without us making judgements or assumptions about their practices. During the data analysis quotes were selected to respectfully represent diverse perspectives.

### Researcher reflexivity

The research team comprised individuals with diverse backgrounds and expertise relevant to PD. The researcher M.R. is a British man with Pakistani heritage in his late twenties whose personal experience of a parent with Parkinson’s has shaped his understanding of the disease within minority ethnic communities. Another member in the analysis and interpretation included M.T., an MSc student of Pakistani background, J.S., a Professor of Ageing and Clinical Psychology with expertise in qualitative research who contributed to the methodology and psychological expertise and A.S., a leading academic and clinical expert in PD who provided guidance and supervision in the design, conduct, analysis and writing.

Aware of how the researcher’s backgrounds could influence the interpretation of data the team engaged in ongoing reflexive practices. M.R. engaged in reflexive practices throughout the study. These included keeping a reflexive journal for self-reflection and critically reflecting on any potential biases. Emerging themes were discussed with co-authors to ensure alternative interpretations were considered. The research team had no prior relationship with the participants.

## Results

A total of 21 individuals who self-identified as belonging to an EM group and were diagnosed with PD participated in this study. The sample included individuals from South Asian (Indian, Pakistani, Sri Lankan), Arab or of mixed ethnic backgrounds. Participants’ ages ranged from 31–71 years (mean age 54 years), and 62% were male (38% female). Five participants were accompanied by their carers, who also contributed to the discussions.

**Figure 1:**
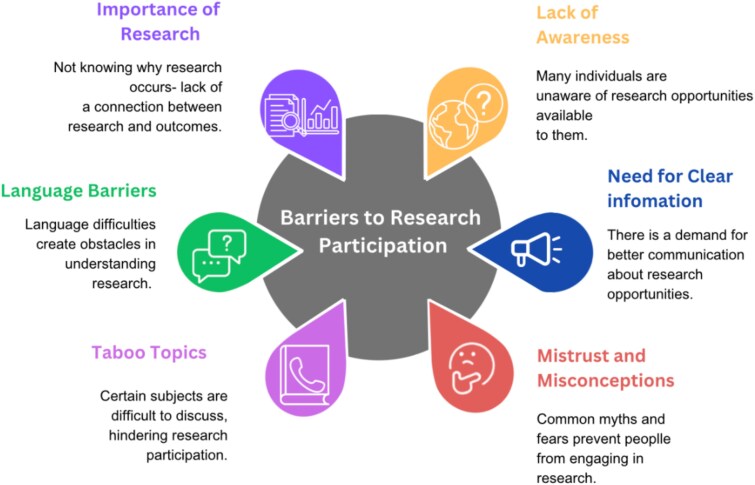
Illustrates the main barriers identified in our study.

Responses were grouped into five themes, which are summarised in Table 2: (i) Lack of awareness of research opportunities; (ii) Lack of trust; (iii) Understanding the Importance and content of Research; (iv) Parkinson’s related and Practical barriers; and (v) Facilitators to Participation. The main barriers identified in our study are shown in Figure 1.

**Table 2:** Overview of the themes.

Theme	Subtheme	Sample quote
Lack of Awareness of research opportunities	Lack of Awareness and Knowledge of Research Opportunities	*‘Not knowing about it in the first place, that there is such research opportunities.’* (P13) (PwP with carer)
	Suggested solutions to improve Awareness of research opportunities	*‘More support, more information, more and like, easy for me to contact someone’* (P4)*.*
Lack of trust	Suspicion and Misconceptions about Research	*‘They believe in lots of myths’* (P3)
	Taboo Topics and Communication Barriers	*‘The hallucinations, that's very difficult to talk about with people […] They’re more personal and probably hidden [. . .] therefore you try and keep it quiet about it.’* (P13) (PwP with carer)
Understanding the Importance and Content of Research	Understanding the Importance and Methods of Research and faith in research	*‘In the past, it wasn’t clear to me why it was important to take part in research.’* (P1)
	Topics of Interest in Research	*‘I’d like to see more research on non-motor symptoms and the psychological aspects of Parkinson’s, including anxiety.’* (P6)
Parkinson’s related and practical barriers Facilitators to Participation	Parkinson’s related barriers	*‘Sometimes it’s apathy because the questionnaires are so long. If you’d sent me this questionnaire, I would not have done it.’* (P2)
	Financial, time and Logistical barriers to accessing research studies	*‘If they have to travel a long distance [. . .] that will stop them from involving in the research.’* (P3)
	Preference for Remote and Flexible Research Methods	*‘I would prefer flexibility [. . .] this sort of thing is far easier for me than going to a hospital.’* (P1)

### Lack of awareness of research opportunities

#### Lack of awareness and knowledge of research opportunities

Many participants mentioned lack of awareness about available research opportunities. In particular, participants reported little discussion of research in their communities and minimal experience of research exposure. However, this was not the case for all and some participants reported being approached by targeted recruitment strategies.

‘Not knowing about it in the first place, that there is such research opportunities.’ (P13) (PwP with carer)

‘People need to understand research can just be like this, question-based research as well, and I don’t think that’s explained efficiently and well to people of the first generation immigrants. And that’s what we need to get across.’ (P2)

#### Suggested solutions to improve awareness of research opportunities

Some participants mentioned better written or verbal information and outreach dissemination was needed:

‘There needs to be a bigger shout-out [...] you’ve got to reach out to individuals.’ (P1)

‘like you approached us, I wouldn’t know that opportunity to take part in this research was there. If you hadn’t approached me, for example.’ (P13) (PwP with carer)

‘More support, more information, more and like, easy for me to contact someone’ (P4).

### Lack of trust

#### Suspicion and misconceptions about research

For many mistrust and misconceptions were common: these included ideas that research couldn’t be questionnaire or interview based, and beliefs that as someone of EM you might be being used as a ‘guinea pig’(P3) before the researched product was used on other people.

‘They believe in lots of myths [...] that kind of anxiety and worry will prevent them joining in research.’ (P3)

‘People have a belief that if you are being a Guinea pig and then you will be a different person after, then if you’re taking part in the research. Such kind of myths and beliefs still existing.’ (P3)

‘There’s a lot of suspicion in our parents’ generation first generation. What is research? A lot of people are seeing that research will be taking a medication and it would be like a huge experiment and they'll be experimented on.’ (P2)

‘People sometimes think that research want to try something on an ethnicity before it releases to the world [...] like a lab rat or people just testing anything.’ (P6)

#### Taboo topics and communication barriers

Stigma was also a key barrier, particularly surrounding sensitive health topics. There was a particular reluctance to discuss certain ‘difficult’ symptoms like non motor symptoms, particularly regarding mental health.

‘The hallucinations, that’s very difficult to talk about with people.’ (P13) (PwP with carer)

‘They’re more personal and probably hidden […] therefore you try and keep it quiet about it.’ (P13) (PwP with carer)

For some language difficulties posed significant barriers:

‘Language is a barrier [...] people from the first generation would find it difficult unless they had a younger person there to help them’ (P2).

### Understanding the importance and content of research

#### Understanding the importance and methods of research and faith in research

Many understood to an extent the reason for research is to generate new knowledge and new treatments and approaches, however others did not understand this,

‘In the past, it wasn't clear to me why it was important to take part in research.’ (P1)

Further some had lost faith in research:

‘I want somebody to find a cure like vaccine they found so quickly, why Parkinson’s taking so long time they have 2014 it’s been 10 years I been hearing this research, that research they keep failing.’ (P19)

#### Topics of interest in research

Some thought of research only as studies to find a cure for Parkinson’s. However, others wished for research on specific symptoms that are relevant to them:

*‘Well, we thought research was when they're trying to find a cure for the Parkinson.’* (P14)

‘I’d like to see more research on non-motor symptoms and the psychological aspects of Parkinson’s, including anxiety.’ (P6)

‘I think they should focus on improving balance issues.’ (P8)

‘I think more research is needed in service provision for those in Ethnic Minorities.’ (P2)

‘I wish they would look beyond chemical interventions.’ (P7)

### Parkinson’s related and practical barriers

#### Parkinson’s related barriers

Parkinson’s symptoms themselves were barriers to participation, particularly fatigue and apathy were mentioned. ‘Off-periods’ and other symptoms such as anxiety were also obstacles.

‘Sometimes it’s apathy because the questionnaires are so long. If you’d sent me this questionnaire, I wouldn’t have done it.’ (P2)

‘Last time I couldn’t do what you wanted me to do because I was very anxious.’ (P5)

‘I think anxiety some people might be anxious about taking part in research […] for us […] maybe anxiety about taking part in research.’ (P13) (PwP with carer)

#### Financial, time and logistical barriers to accessing research studies

Others mentioned practical challenges as obstacles to participation including travel, time off work and finances.

‘If they have to travel a long distance [...] that will stop them from involving in the research.’ (P3)

‘Accessibility when I was working full-time [...] being able to go once a week to take part was difficult.’ (P1)

‘Financial barriers is the only thing I can think of.’ (P18)

### Facilitators to participation

#### Preference for remote and flexible research methods

As a results, flexibility in mode of research participation was important to many:

‘I would prefer flexibility [...] this sort of thing is far easier for me than going to a hospital.’ (P1)

‘I prefer video calls [...] going somewhere is very difficult.’ (P14) (PwP with carer)

## Discussion

Our analysis revealed that research participation amongst EM PwP are influenced by a complex interplay of awareness, trust, communication barriers, understanding of research and practical constraints but also highlights some facilitators to increased research participation.

### Novel contributions to understanding Parkinson’s disease research participation barriers

This study provides unique insights specific to PD research amongst ethnic minorities. While a number of barriers to research participation amongst ethnic minorities have been documented across various health conditions mentioned below, this study is the first UK-based qualitative study to examine the barriers to research participation amongst predominantly South Asian and Arab people living with PD.

However, our study is it identified several new PD-specific barriers. We uniquely documented how stigma around discussing nonmotor symptoms, particularly hallucinations and mental health aspect creates communication barriers that may affect research participation. While participants expressed interest in such research (‘I’d like to see more research on non-motor symptoms’), they simultaneously described these symptoms as ‘very difficult to talk about’ and ‘more personal and probably hidden’, creating a paradox where most-needed research topics are hardest to discuss. Second, the unpredictable nature of motor fluctuations and ‘off-period’ fatigue and anxiety emerged as a previously undocumented research-specific barriers, requiring unique accommodation strategies unlike stable chronic disease patterns. Finally, our study’s focus on research-naive EM populations (most participants had no prior research experience) provides important insights into lived experiences of barriers that both prevented previous research engagement and were encountered during participation, as well as ongoing Parkinson’s-specific challenges that affect research engagement. Participants described real obstacles they faced when deciding to join our study and genuine barriers that had previously deterred them from research, including symptom-related challenges (‘Sometimes it’s apathy because the questionnaires are so long’) during the study, as well as practical barriers and communication difficulties. They experienced barriers show that EM Parkinson’s populations face genuine obstacles before and during research engagement, with important implications for inclusive research design.

### Learning from successful Parkinson’s disease studies with higher ethnic minority participation

Previous studies in PD that were able to recruit higher proportions of ethnic minorities used targeted efforts to improve diversity. The East London Parkinson’s Disease Project achieved remarkable diversity (39% South Asian, 44% White, 11% Black) through extensive community outreach, recruitment in community settings rather than hospitals, culturally appropriate materials used a diverse multilingual research team. In addition, recruitment was carried out by a team that mirrored the ethnic and linguistic diversity of the local community, which helped build trust and provided culturally sensitive communication. Furthermore, the study team employed flexible participation options and translated study materials. STEADY-PD III demonstrated that large-scale trials can improve diversity through mandatory 10% minority recruitment targets, supplemental funding for diversity recruitment and strategic site selection with community partnerships. The FIRE-UP PD study involved community members as co-researchers and developed culturally tailored educational materials to address barriers. These successful strategies align with our findings. Our participants emphasis on a ‘bigger shout-out’ mirrors the East London Project’s community-centred approach, while preference for ‘insider’ researchers reflects FIRE-UP PD’s community co-researcher model. Our proposed framework of strategies is detailed in Table 3. However, our deeper exploration of cultural taboos around nonmotor symptoms suggests successful recruitment requires even more complex cultural awareness.

**Table 3:** Strategies to improve research participation amongst ethnic minorities.

Key focus area	Implications
**Building trust**	Highlight transparency in research objectives, participation processes and methodology. This will allow participants to fully understand their involvement. Employ researchers from their community backgrounds to help open dialogue.
**Improving communication**	Provide clear information about research using plain language and avoiding complex medical terminology. Use multiple communication channels, including social media and preferred types of contact (e.g. phone calls, direct messages).
**Addressing practical barriers**	Offer financial support for travel and virtual visits. Prioritise remote and flexible research methods to accommodate the needs of PwP.
**Personalised research**	Highlight the relevance of research outcomes to participant’s needs and interests. Clearly link research participation to better outcomes. Focus on non-motor symptoms and alternative approaches.
**Recruitment communication and awareness**	Address the reasons participants may lose interest, such as perceptions that research is irrelevant or not beneficial. Use clear messaging to demonstrate how participation directly impacts outcomes and benefits the wider PD community.

**Table 4:** Recruitment sources.

Recruitment source	N
Neurology clinics	1
Facebook—Parkinson’s groups	10
Instagram	2
Community centres	3
Snowballing (Referring others from EM backgrounds who might be interested in the study.)	5

The barriers to research found in this study overlap with findings in other disease groups and their ability to recruit persons from diverse backgrounds. In dementia/Alzheimer’s research, studies document similar barriers, including mistrust, fear of adverse effects of research procedures [17], language and communication barriers, unfamiliarity with research terminology [18], practical and logistical barriers [17], geographic isolation and limited access to research centres [19], and cultural beliefs and stigma around mental health and dementia [18].

### Awareness and engagement

We identified a significant lack of awareness about research opportunities amongst PwP from EM groups. This lack of awareness is likely contributed to but not solely due to insufficient outreach efforts. Many participants had been directly approached through targeted recruitment strategies, and poor understanding of the content of research likely contributed to this perception of poor availability of research. Other factors such a mistrust, cultural stigma, language challenges and low understanding of the importance of research may have created disengagement from research opportunities despite their availability. Our experience showed that tailored recruitment is important. The initial recruitment through neurology clinics yielded very little uptake, potentially influenced by limited clinician and researcher time in outpatient or office settings to explore barriers. Such barriers may be greater for EM participants, highlighting the need to shift outreach strategies to community centres, support groups, social media and word-of-mouth referrals. This strategy increased participation in our study, consistent with evidence from a previous Parkinson’s study in a Hispanic population, which highlighted that community involvement and engagement were shown to significantly increase participation amongst minority groups [20]. These findings also align with recent research by Sanchez *et al.* [13], who created a comprehensive dashboard of barriers to research participation in their FIRE-UP PD study. They found that infrastructure barriers (including lack of digital access) and language barriers and lack of awareness were primary barriers to engagement for underrepresented populations in Parkinson’s disease research. Their study similarly concluded that community-centred recruitment approaches were important to overcome these barriers. Additionally, Tilley *et al*. [21] demonstrated that community based research frameworks significantly improved recruitment of minorities into clinical trials by addressing awareness and engagement barriers through local partnerships. Such programmes should focus on raising awareness about the purpose and safety of research and inclusive recruitment practices.

### Trust and cultural barriers

Medical mistrust represents a significant barrier to research participation amongst EM communities, including perceived discrimination and disparities in care [22]. In our study, we found that mistrust and misconceptions about research were widespread amongst PwP from EM groups, with fears reflecting uncertainty about the scope and safety of research. This aligns with Subramanian *et al.* [23], who demonstrated that mistrust can significantly delay diagnosis and treatment engagement in PD amongst EM communities. Cultural context and stigma create additional obstacles to research participation, particularly regarding nonmotor symptoms and mental health symptoms aspects of PD. Another study highlighted how cultural beliefs about illness and treatment in EM populations with PD may differ substantially from majority populations [24], influencing which symptoms are acknowledged and discussed. This cultural stigma together with misconceptions about research methods and poor knowledge of Parkinsons, can significantly worsen disengagement from research opportunities. Community engagement may not only allow for reach of wider populations but also reduce mistrust and stigma. This was demonstrated by previous research showing that engagement with trusted community leaders and community health workers can help address stigma and improve participation amongst EM PwP groups in PD research [25].

### Practical and Parkinson’s-related barriers

Disease-specific and socioeconomic factors create significant barriers to research participation amongst EM populations with PD. Another study found that severe motor symptoms prevented research participation [26], mirroring our participant’s need to prioritise limited periods of ‘on time’ for essential activities. Speech and mobility impairments [27], compound these challenges, particularly when combined with lower socioeconomic status, which linked to reduced healthcare access [28]. These combined challenges of travel, financial constraints and language difficulties, may translate into reduced research access, leading to the underrepresentation and understudy of EM PwP. Some solutions include flexible remote participation options [25] and scheduling around medication timing, which Tilley *et al.* [21] demonstrated increased participation by 37% amongst underrepresented populations.

### Facilitators to participation

The strong preference for flexibility and remote participation reflects how EM PwP are actively seeking research engagement methods that accommodate their specific needs. This finding suggests that traditional research protocols may be exclusionary and that technology enabled participation could be a key strategy for improving representation. A previous study found that remote participation in PD research is feasible and well-received with potential to reduce burden and increase reach this may be beneficial for improving representation in underrepresented groups like EM PwP [29].

### Strengths and limitations

Our findings are overall consistent with existing literature on research participation amongst minority populations with other conditions, including dementia and Alzheimer’s disease, where minority participants experience similar barriers of lack of information on research procedures, mistrust and logistical burdens such as transportation and financial constraints [30]. However, this study provides new specific insights into the experiences of PwP from EM groups in the UK context. A key strength of this study is its outreach through community channels and its focus on a group of EM participants, an often underrepresented group in PD research (many of whom had not previously engaged with research). The qualitative approach allowed for an in-depth exploration of participants experiences and perceptions enhancing the validity of findings. The involvement of a community insider researcher helped build trust and open discussion.

However, the study has some limitations. The sample size while adequate for qualitative research, could not capture all possible experiences within a range of diverse EM groups. Also, as the vast majority of our sample were South Asia and Arab participants the transferability of findings is limited and primarily applicable to these groups. Although many participants had not previously engaged with research, our final sample potentially represents individuals more open to research participation than the broader population of PwP from ethnic minorities.

These limitations highlight the need for caution when generalising findings and demonstrate the importance of further research with diverse ethnic groups. Future research with more diverse groups is needed to confirm these findings.

### Unanswered questions and future research

Several important questions remain for future research. First, intervention studies are needed to test whether implementing the strategies identified in this study actually increases research participation amongst EM PwP populations. Secondly more diverse enrolment is needed to understand disease mechanisms and progression across ethnic groups.

Future studies should also explore the experiences of EM groups not represented in our sample and investigate how intersecting factors such as socioeconomic status, education level and geographic location influence research participation. Long-term studies could show if attitudes change after specific efforts to boost participation. Additionally, research examining the perspectives of healthcare providers and researchers could identify systemic barriers and facilitators to inclusive recruitment practices.

### International transferability and contextual considerations

Our UK findings can help other countries, but they need to be adapted for local contexts. Similar barriers exist everywhere, mistrust of research, language problems and practical difficulties like travel and cost. However, there are some differences between countries, e.g. unlike in the UK in the US laws require minority inclusion in trials (leading to better participation); different minority groups live in each country; and healthcare systems work differently.

## Conclusion

This study identified multiple interrelated barriers to research participation amongst EM PwP in the UK, including limited awareness, mistrust, practical limitations and insufficiently personalised research approaches. We also identified important facilitators, particularly flexible participation options and community-based recruitment strategies. Addressing these barriers and using facilitators requires research approaches that consider the specific needs of different ethnic groups. Implementation of targeted strategies to enhance inclusivity could improve the representativeness of PD research.

## Supplementary Material

aa-25-0840-File002_afaf296

## Data Availability

Due to the nature of this qualitative study and the sensitive, identifiable content shared by participants. The full interview data cannot be made publicly available. Excerpts supporting the findings are included within the article. Further information may be available upon reasonable request to the corresponding author subject to ethical approval.
