# Correction to: Participation of ethnic minorities in Parkinson’s research: challenges and needs. A qualitative study

**DOI:** 10.1093/ageing/afag001

**Published:** 2026-02-19

This is a correction to: Mouhammed Ramadhan, Mahil Tufail, Joshua Stott, Anette Schrag, Participation of ethnic minorities in Parkinson’s research: challenges and needs. A qualitative study, *Age and Ageing*, Volume 54, Issue 10, October 2025, afaf296, https://doi.org/10.1093/ageing/afaf296

The following changes have been made to the originally published manuscript. The order of authors has been emended to read: “Mouhammed Ramadhan, Mahil Tufail, Joshua Stott, Anette Schrag” instead of: “Mouhammed Ramadhan, Anette Schrag, Mahil Tufail, Joshua Stott”.

