# Supplementary material for: Participation of ethnic minorities in Parkinson’s research: challenges and needs. A qualitative study
Source: Age Ageing. 2025 Oct 24;54(10):afaf296. doi: 10.1093/ageing/afaf296 (PMC12551381; doi:10.1093/ageing/afaf296)
Supplement: aa-25-0840-File002_afaf296 [file aa-25-0840-file002_afaf296.docx]

**Participation of Ethnic Minorities in Parkinson's Research: Challenges and Needs. A qualitative study**

**Appendix 2- Interview questions on barriers to research**

| (i) Have you ever participated in any research before? |
| --- |
| (ii) What do you think research is for and what do you think research involves? |
| (iii) What type of research do you feel is needed in PD, particularly for your population group. |
| (v) If you have not participated in research, what has prevented you from accessing research?  (iv) In research what type of research would you like to be involved in? |
| (vi) Would you be comfortable doing research with remote means? |

**Interview Guide Development**

The semi-structured interview guide was adapted from a previous study incorporating patient and public involvement (PPI) input, with substantial modifications for ethnic minority participants. This interview was part of a larger study examining multiple aspects of Parkinson's disease experiences among ethnic minorities. For this research participation component, we added specific questions addressing barriers and facilitators to research engagement among ethnic minority populations.

Key ethnic minority adaptations included culturally sensitive language choices ("worry" vs "anxiety"), questions exploring how cultural/religious backgrounds affect healthcare/research experiences, assessment of language barriers, and investigation of cultural taboos around health topics.

Through an iterative development process involving team discussion and refinement, the research participation section explored previous research experience, understanding of medical research, perceived barriers to participation, cultural considerations affecting research decisions, research interests and needs and preferences for remote participation methods. The research team included members with diverse ethnic backgrounds and community engagement experience.
